# Supplementary material for: Genetic and economic efficiencies of alternative breeding schemes for improvement of local breeds in low-input production systems: The case of the Farta sheep in Northwest Ethiopia
Source: PLoS One. 2025 Sep 5;20(9):e0331701. doi: 10.1371/journal.pone.0331701 (PMC12412953; doi:10.1371/journal.pone.0331701)
Supplement: Attachment — Submitted filename: Recommendation.docx [file pone.0331701.s002.docx]

Review

Genetic and Economic Efficiencies of Alternative Breeding Schemes for Improvement of Local Breeds in Low-input Production Systems

**Introduction**

- Emphasize why Farta sheep is chosen by mentioning their socio- economic or ecological importance
- It should be explained why selection is performed on male?

**Material and Methods**

- Consider adding a country map highlighting the study area, and also add an image of Farta Sheep breed

Minor comments

- There is some repetition and minor grammatical issues

Strength

The manuscript is well structured with a logical flow of ideas. Citations are properly used to support key points. Breeding schemes are clearly explained with practical considerations. It sets the context by highlighting the importance of livestock in Ethiopia and provides adequate background on Farta sheep.
