# Supplementary material for: Genetic and economic efficiencies of alternative breeding schemes for improvement of local breeds in low-input production systems: The case of the Farta sheep in Northwest Ethiopia
Source: PLoS One. 2025 Sep 5;20(9):e0331701. doi: 10.1371/journal.pone.0331701 (PMC12412953; doi:10.1371/journal.pone.0331701)
Supplement: Attachment — Submitted filename: PONE-D-25-28186.docx [file pone.0331701.s003.docx]

In the present study "Genetic and Economic Efficiencies of Alternative Breeding Schemes for Improvement of Local Breeds in Low-input Production Systems: The Case of the Farta Sheep in Northwest Ethiopia. The manuscript investigates sustainable genetic improvement in low-input livestock systems, focusing on indigenous breed, Farta sheep. The research explores the efficiencies of three alternative breeding programs to identify appropriate schemes that can sustainably enhance sheep productivity within existing breeding practices and infrastructure.

**Major comments:**

- A key aspect of the study involves deriving genetic and phenotypic parameters for body weights from Farta or crossbred sheep. However, it is important to note that parameters for lambing rate (NLW) and fertility were adapted from studies on non-Ethiopian breeds, which may not accurately represent Ethiopian indigenous sheep in low-input systems.
- Scheme II is presented as a suitable option for the genetic improvement of indigenous sheep in low-input systems due to its genetic and economic benefits, as well as its operational feasibility. However, the study acknowledges that the operational feasibility from a smallholder farmer's perspective in the Ethiopian context is not extensively detailed. Challenges such as controlled mating and centralized ram selection are difficult to implement given traditional communal grazing and multi-sire mating practices prevalent in Ethiopia.
- While farmers' trait preferences are mentioned, the breeding objective does not fully explore local definitions of "good mothering ability" and culturally significant traits such as tail type and color^.^

**Minor comments:**

- Consider adding "community-based breeding" to the keywords.
- The document contains long paragraphs that could be broken down for improved readability.
